# Apatite/Chitosan Composites Formed by Cold Sintering for Drug Delivery and Bone Tissue Engineering Applications

**DOI:** 10.3390/nano14050441

**Published:** 2024-02-28

**Authors:** Anna Galotta, Öznur Demir, Olivier Marsan, Vincenzo M. Sglavo, Dagnija Loca, Christèle Combes, Janis Locs

**Affiliations:** 1 Department of Industrial Engineering, University of Trento, Via Sommarive 9, 38123 Trento, Italy; vincenzo.sglavo@unitn.it; 2 Institute of Biomaterials and Bioengineering, Faculty of Natural Sciences and Technology, Riga Technical University, Pulka St. 3, LV-1007 Riga, Latvia; oznur.demir@rtu.lv (Ö.D.); dagnija.loca@rtu.lv (D.L.); janis.locs@rtu.lv (J.L.); 3 Baltic Biomaterials Centre of Excellence, Riga Technical University, Pulka St. 3, LV-1007 Riga, Latvia; 4 CIRIMAT, Toulouse INP, Université Toulouse 3 Paul Sabatier, CNRS, Université de Toulouse, ENSIACET, 4 Allée Emile Monso, BP 44362, CEDEX 4, 31030 Toulouse, France; olivier.marsan@toulouse-inp.fr (O.M.); christele.combes@ensiacet.fr (C.C.)

**Keywords:** nanocrystalline apatite, chitosan, apatite/chitosan composites, strontium ranelate, mussel shells, cold sintering, dissolution–precipitation synthesis, drug delivery

## Abstract

In the biomedical field, nanocrystalline hydroxyapatite is still one of the most attractive candidates as a bone substitute material due to its analogies with native bone mineral features regarding chemical composition, bioactivity and osteoconductivity. Ion substitution and low crystallinity are also fundamental characteristics of bone apatite, making it metastable, bioresorbable and reactive. In the present work, biomimetic apatite and apatite/chitosan composites were produced by dissolution–precipitation synthesis, using mussel shells as a calcium biogenic source. With an eye on possible bone reconstruction and drug delivery applications, apatite/chitosan composites were loaded with strontium ranelate, an antiosteoporotic drug. Due to the metastability and temperature sensitivity of the produced composites, sintering could be carried out by conventional methods, and therefore, cold sintering was selected for the densification of the materials. The composites were consolidated up to ~90% relative density by applying a uniaxial pressure up to 1.5 GPa at room temperature for 10 min. Both the synthesised powders and cold-sintered samples were characterised from a physical and chemical point of view to demonstrate the effective production of biomimetic apatite/chitosan composites from mussel shells and exclude possible structural changes after sintering. Preliminary in vitro tests were also performed, which revealed a sustained release of strontium ranelate for about 19 days and no cytotoxicity towards human osteoblastic-like cells (MG63) exposed up to 72 h to the drug-containing composite extract.

## 1. Introduction

Hydroxyapatite (HAp) is one of the most studied materials in the biomedical field, especially in the frame of bone tissue engineering applications such as implants, fillers, scaffolds, coatings, cements and drug delivering devices [1,2,3,4,5]. HAp is an appealing candidate as a synthetic bone substitute due to its biocompatibility, bioactivity and osteoconductivity but also because of its chemical composition and crystal structure resembling those of natural bone apatite [6,7,8]. However, the in vitro and in vivo performance of pure stoichiometric HAp is hindered by its high chemical stability, poor osteoinduction, limited solubility and resorbability in in vivo conditions [9,10]. Ressler et al. [11] demonstrated that substituted apatite/chitosan composites induce higher phosphate deposition and expression of osteogenesis-related markers with respect to non-substituted apatite/chitosan composites. Indeed, the mineralised part of bone tissue consists of a nanocrystalline, non-stoichiometric, carbonated and multiple ion-substituted apatite [12,13,14]. Carbonates, the major ion substitutes (~3–8 wt% [15,16,17]), and other trace elements (such as Mg^2+^, Na^+^, K^+^, Sr^2+^, Cl^−^, etc.) can be easily accommodated in the crystal lattice of biological apatites, and they play a crucial role in bone tissue functions and enhance the biological properties of bone apatite [10,18]. Moreover, the high surface reactivity of bone apatite is associated with the presence of a thin (few nanometres) hydrated layer on the surface of apatite nanocrystals, which mostly contains Ca^2+^, CO_3_^2−^ and HPO_4_^2−^ ions and is involved in homeostasis as well as the regulation of ionic concentration in body fluids [14,19,20,21].

HAp is often combined with biopolymers, such as collagen [22,23], silk fibroin [24,25], poly-L-lactic acid [26,27], chitosan [28,29], etc., in order to better mimic the complexity of bone tissue and to enhance the mechanical properties and biological response. For example, the bioactivity and osteoconductivity of HAp/poly-L-lactic acid composites were improved by triggering the degradation rate of the material through poly-glycolic acid blending [26]. Nonetheless, Zima [30] reported an improvement in the compressive strength of HAp from 2 MPa to 24 MPa/32 MPa by adding 17 wt%/23 wt% chitosan. Similarly, HAp mechanical performance was strongly improved by the addition of 30 vol% PLA; the compressive strength increased from ~19 MPa to ~81 MPa, while the flexural strength increased to ~32 MPa [31].

Starting from calcium and phosphorous precursors, hydroxyapatite can be produced by several synthesis methods including mechanochemistry [32,33,34], hydrothermal synthesis [35,36,37], wet precipitation and dissolution–precipitation synthesis [38,39,40]; interestingly, the use of biogenic Ca sources has been proven to be an effective strategy to introduce trace elements naturally present in algae, seashells, snail shells, eggshells, and mammalian and fish bones [41,42,43,44,45,46]. In the present work, mussel shells were used as natural calcium carbonate source to produce bone-like apatite powders by dissolution–precipitation synthesis. Mussel shells are known to contain Mg^2+^, Sr^2+^, Na^+^ and K^+^ in variable trace amounts depending on the species, geographical and environmental conditions [47,48,49,50]. Dissolution–precipitation synthesis is a straightforward, versatile, implementable and cost-effective method, which ensures high control over the reaction by continuously monitoring the processing parameters (temperature, pH, stirring time and speed) [51,52]. It is also efficient in producing nanopowders with high specific surface area and tunable crystallinity [51,52,53,54]. The synthesis of calcium phosphates by dissolution–precipitation has already been investigated to produce, for example, B-type carbonated HAp [38], poly-ion-substituted calcium-deficient hydroxyapatite [39] and HAp/chitosan composite in one pot [55]. In the present work, dissolution–precipitation was further explored to synthesise HAp/chitosan composites loaded with antiosteoporotic drug–strontium ranelate (SrRAN). Chitosan is a natural polysaccharide widely used in the biomedical field and is characterised by high biodegradability, bioactivity, antimicrobial and osteoconductive behaviour [28,30,56,57], while SrRAN is an antiosteoporotic agent, able to impair bone turnover by promoting bone formation by osteoblasts while downregulating the bone resorption by osteoclasts [58,59,60,61,62,63]. Chitosan and SrRAN can be combined with mussel shell-derived apatite, to mimic natural bone tissue, tailor the overall mechanical behaviour of the final composite [55], and improve its biological response.

In order to produce mechanically resistant bioceramic components, sintering should be carried out, typically at a temperature of around 1000 °C [64,65,66]. Nevertheless, such temperatures can cause undesired phase transformation and uncontrolled grain growth [65], and loss of osteoconductivity and bioactivity [67,68,69] related to the specific features of bone-like apatite, namely, metastability and reactivity of the superficial hydrated layer, non-stoichiometry and ion-substitution. In the past decades, new sintering approaches have been developed to reduce the sintering temperature and time, such as hot pressing, flash sintering, spark plasma sintering and cold sintering [70,71]. Low temperature sintering methods like cold sintering and spark plasma sintering are of particular interest in the biomedical field since calcium phosphates can be consolidated at a temperature of no more than 300 °C in a few minutes [21,72,73,74,75,76] by the simultaneous application of external pressure, temperature and, occasionally, a transient liquid phase. The remarkable reduction in sintering temperature allows the preservation of the fundamental characteristics of biomimetic apatite and opens up new ways to sinter metastable systems and ceramic-based composites in a single step [69,77,78,79,80]. Recently, Guo et al. [81] reported the cold sintering of chitosan/hydroxyapatite composites up to ~90% relative density, under variable uniaxial pressure (from 200 MPa to 600 MPa), temperature (from 50 °C to 150 °C) and holding time (from 5 min to 90 min). Similar relative density was also reported for amorphous calcium phosphate (ACP) consolidated at room temperature under 1.5 GPa in 10 min [54]. Nonetheless, Hu et al. [31] reported for the first time a drug-loaded HAp/PLA composite produced by cold sintering (500 MPa, 80 °C, 30 min) and investigated the doxorubicin release profile in a saline solution.

The aim of the present work was to develop a novel SrRAN-loaded biomimetic apatite/chitosan composite by dissolution–precipitation synthesis and cold sintering and to investigate whether the produced material could become a possible candidate for local drug delivery and bone tissue engineering purposes. Indeed, cold sintering could become a valuable and promising processing technology to develop drug-carrier ceramic-based systems, considering its fundamental features (i.e., low temperature, short processing time), which make it possible to maintain the integrity of the organic matter in the final product. To the best of the authors’ knowledge, the present study is one of the first examples in which cold sintering is exploited to produce potential drug delivery systems, and in this context SrRAN is used as a model drug for the first time. 

The current research includes an extensive physicochemical characterisation of the produced materials, an investigation of the effects on the composites caused by the pressure applied during cold sintering, determination of the SrRAN release profile and a preliminary cell viability assessment. The present work follows and deepens a previous study on mussel shell-derived HAp/chitosan composites, in which the mechanical properties of the material were investigated [55].

## 2. Materials and Methods

### 2.1. Synthesis and Cold Sintering of HAp and HAp/Chitosan Composites

**Figure 1:**
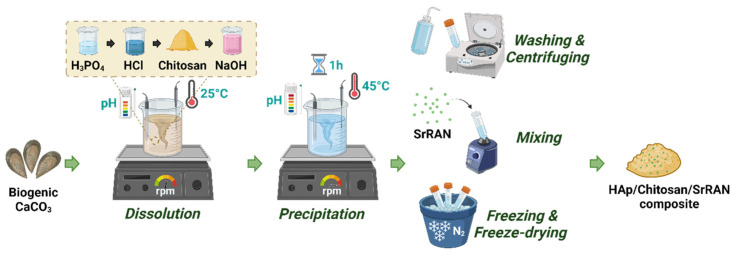
Dissolution–precipitation synthesis of SrRAN-loaded HAp/chitosan composite. The figure was created with Biorender.com.

HAp and HAp/chitosan composites were synthesised by the dissolution–precipitation method, as described in a previous work [55]. Briefly, 10 g of biogenic CaCO_3_ obtained from crushed mussel shells was dispersed in 600 mL of distilled water at room temperature using a Biosan MM-1000 overhead stirrer at 350 rpm. Calcium carbonate was fully dissolved by adding first 12.6 mL of 4.76 M H_3_PO_4_ (75% pure, CAS No. 7664-38-2, Merck KGaA, Darmstadt, Germany) solution and then 64.5 mL of 3 M HCl (37%, CAS No. 7647-01-0, Merck KGaA, Darmstadt, Germany solution. The pH and temperature of the system were constantly monitored using a WTW inoLab pH 7110 digital pH meter and a temperature probe connected to a Biosan MSH-300i hot plate, respectively. Chitosan powder (chitosan from shrimp shells, ≥75% deacetylated, CAS No. 9012-76-4, Merck KGaA, Darmstadt, Germany) was then added to the solution (10 wt% with respect to HAp). The stirring rate was increased to 600 rpm and 96 mL of 2 M NaOH (pellets for analysis, CAS No. 1310-73-2, Merck KGaA, Darmstadt, Germany) solution were rapidly added to induce the precipitation of HAp and HAp/chitosan. The system was then heated up to 45 °C and continuously stirred for 1 h at 600 rpm. Eventually, the obtained slurry was repeatedly centrifuged and washed with distilled water to eliminate all NaCl residues and lyophilised for 72 h (in a BETA 2-8 LSC plus Martin Christ Freeze Dryer, Osterode, Germany). After washing, a batch of HAp/chitosan composite slurry was further loaded with SrRAN (CAS No. 135459-87-9, Zhishang Industry Co., Ltd., Jinan, China) by mixing 5 wt% SrRAN powder in a high-speed mixer (DAC 150.1 FVZ-K Speed Mixed^®^ by Hauschild GMBH & CO.KG, Hamm, Germany) for 2 min at 3500 rpm. The drug-loaded slurry was then freeze-dried for 72 h. A simplified scheme of the process is shown in Figure 1. The produced powders were labelled as HAp, HAp10Chit and HAp10ChitSrRAN.

Pellets (13 mm diameter) were produced by cold sintering at room temperature by uniaxial pressing 0.4 g of powder into a cylindrical die under 250 MPa to 1500 MPa for 10 min, as described in [55]. In addition, 6 mm diameter pellets were also produced for the in vitro tests by cold sintering 0.08 g of powder under 1000 MPa at room temperature for 10 min.

### 2.2. Physicochemical Characterisation 

The synthesised powders and bulk components were analysed by X-ray diffraction (XRD) using a Malvern PANalytical (Worcestershire, UK) Aeris set at 40 kV and 30 mA and equipped with a Cu/Kα (*λ* = 1.5406 Å) radiation source. The XRD patterns were then elaborated by X’PertHighScore software (Malvern Panalytical, Worcestershire, UK) and compared with reference patterns from the International Centre for Diffraction Data (ICDD^®^) database. The average crystallite size (*D_hkl_*) was estimated by the Scherrer formula in Equation (1):

(1) $$D_{hkl}=0.94\lambda /(\beta \cdot \cos\theta_{hkl})$$

where *λ* is the X-ray wavelength, *θ_hkl_* is the diffraction angle corresponding to the ‘*hkl*’ plane and *β* is the width at half height of the corresponding ‘*hkl*’ peak. A fully crystalline stoichiometric hydroxyapatite provided by Marion Technologies was also analysed and used as a reference to determine *β*, according to Equation (2):

(2) $$\beta =\sqrt{{∆}_{S}^{2}-{∆}_{R}^{2}}$$

where Δ*_S_* and Δ*_R_* are the full width at half height of the analysed sample and of the reference, respectively. 

The specific surface area (SSA) of the powder was determined by the Brunauer–Emmett–Teller (BET) method, using a Quantachrome (Boyton Beach, FL, USA) Monosorb MS-21 monopoint analyser. The analysis was carried out by degassing 40 mg of powder at room temperature for about 1 h with a Quantachrome (Boyton Beach, FL, USA) Autosorb Degasser and then proceeding with the nitrogen adsorption measurements at −196 °C. The measurements were performed in triplicate. Starting from the SSA value, the average spherical particle size (*D*) was estimated according to Equation (3):

(3) D=6/(SSA ρ)

where *ρ* is the powder real density determined by a Quantachrome (Boyton Beach, FL, USA) Micro UltraPyc™ 1200e pycnometer. The density measurement was stopped when the standard deviation of five subsequent runs was smaller than 0.1%, and the measurements were repeated in triplicate for each sample.

The molecular structure and the functional groups of the produced powders and cold-sintered pellets were investigated by Fourier transformed infrared (FTIR) and Raman spectroscopy techniques. FTIR analysis was carried out with a Thermo Fisher Scientific (Waltham, MA, USA) Nicolet iS50 FT-IR spectrometer set on transmission mode from 4000 cm^−1^ to 400 cm^−1^ (64 scans, 4 cm^−1^ scan resolution). Prior to analysis, the specimens were mixed with potassium bromide powder (KBr, CAS 7758-02-3, Sigma Aldrich, St. Louis, MO, USA) and pressed to form a pellet. Once collected, the spectra were processed with OMNIC 9.6.251 software by Thermo Fisher Scientific Inc. (Waltham, MA, USA). Raman spectroscopy was performed with a RAMAN LabRAM HR 800 confocal microscope (Horiba Jobin Yvon, Kyoto, Japan). The sample was directly placed on a slide and continuously irradiated with a gas laser at 633 nm to avoid fluorescence under an Olympus (Shinjuku, Tokyo, Japan) BX 41 microscope, and scanned with an objective ×100 and a numerical aperture of 0.9, corresponding to a lateral and axial resolution of 0.86 μm and 3.13 μm, respectively. The spectra were acquired with a 600 lines/mm grating and a spatial resolution of 2 cm^−1^, and processed with LabSpec 5 software afterwards. Furthermore, a DUOScan^TM^ imaging system for mapping was used to verify the homogeneity of the sample. In particular, an area of 50 μm × 50 μm was investigated per each sample. 

The elemental analysis was determined by atomic absorption spectroscopy (AAS) using an iCE 3000 series AA Spectrometer (Thermo Fisher Scientific Inc., Waltham, MA, USA). In particular, a quantitative analysis was carried out to determine the relative amounts of Ca, Mg, Na and Sr in the powders. For each element, a calibration curve was first constructed using standard solutions prepared from Ca, Mg, Na and Sr reference standards by SCP Science (Baie-D’Urfe, Quebec, QC, Canada). The blank solution was prepared with ultrapure water mixed with 1 vol% nitric acid (69% HNO_3_, CAS 7697-37-2, VWR-BDH Chemicals, Radnor, PA, USA), 0.5 vol% of lanthanum nitrate (La(NO_3_)_3_, SCP Science, Baie-D’Urfe, Quebec, QC, Canada) and 0.5 vol% cesium chloride (CsCl, SCP Science, Baie-D’Urfe, Quebec, QC, Canada). The samples were prepared for the analysis by dissolving 100 mg of powder in a 100 mL flask, with the addition of 5 mL HNO_3_ acid and ultrapure water up to 100 mL volume. The matrix modifiers (1 vol% HNO_3_, 0.5 vol% La(NO_3_)_3_ and 0.5 vol% CsCl) were also added to the sample solutions before the analysis.

The carbonate content of the samples was evaluated in triplicate using a CO_2_ coulometer (UIC CM 5014 coulometer, Coulometrics, Fort Collins, CO, USA) that measures in a closed circuit the CO_2_ released during sample dissolution in an acid solution (2 M HClO_4_). 

The total phosphorous content in the form of PO_4_^3−^ and HPO_4_^2−^ in the synthesised powders was determined using visible spectrophotometry by analysing the phosphor-vanado molybdenum complex at 460 nm with a Shimadzu UV-1800 spectrophotometer (Shimadzu Corporation, Kyoto, Japan). However, such a method has the limitation that it is not possible to directly discriminate the phosphorous involved in PO_4_^3−^ ions from that in HPO_4_^2−^ ions. Thus, to overcome this limitation and determine the HPO_4_ content, the samples (~100 mg) were heat-treated at 600 °C for 30 min to form pyrophosphate [82] according to the reaction in Equation (4):

(4) $$2HPO_{4}^{2-}\to {P_{2}O_{7}}^{4-}+H_{2}O$$

After calcination, the powder was dissolved in 6 M HClO_4_ solution and the resulting solution was split into two daughter solutions. The first daughter solution was immediately titrated with a colourimetric indicator to determine only the PO_4_^3−^ content. The colourimetric indicator consisted of ammonium heptamolybdate tetrahydrate solution (80 g/L) and ammonium monovanadate solution (4 g/L) in equal volume. Conversely, the second daughter solution was titrated with the colourimetric indicator after undergoing hydrolysis for 1 h at 100 °C to induce the hydrolysis of P_2_O_7_^4−^ ions (reverse reaction of Equation (4)) and thus determine the total PO_4_^3−^ + HPO_4_^2−^ content. Therefore, the amount of HPO_4_^2−^ ions was determined from the absorbance at 460 nm of the two daughter solutions (total phosphorous in PO_4_^3−^ + HPO_4_^2−^ and PO_4_^3−^ only), as in Equation (5):

(5) $$\left[HPO_{4}^{2-}\right]=\left[{HPO_{4}}^{2-}+{PO_{4}}^{3-}\right]-[{PO_{4}}^{3-}]$$

Nonetheless, the HPO_4_^2−^ content could be underestimated due to a side reaction which might occur in the presence of carbonate ions according to the reaction in Equation (6):

(6) $$2HPO_{4}^{2-}+{CO_{3}}^{2-}\to 2{PO_{4}}^{3-}+CO_{2}+H_{2}O$$

The thermal behaviour of the produced powders and crushed cold-sintered materials was investigated by thermogravimetric analysis (TGA) combined with differential scanning calorimetry (DSC), using a Mettler Toledo (Columbus, OH, USA) TGA/DSC 3+, STARe System thermobalance. A 10–15 mg sample was added to an alumina crucible and heated up to 1200 °C in air flow with 10 °C/min heating rate. 

The morphology of the powders and cold-sintered pellets was investigated by field emission scanning electron microscopy (FE-SEM) with a MEB-FEG JEOL (Akishima, Tokyo, Japan) JSM 7800F Prime–EDS microscope. The samples were coated with carbon prior being observed at the microscope. 

The bulk density of cold-sintered pellets was estimated by the geometrical method considering the weight, the thickness and the diameter of the specimens. 

### 2.3. In Vitro Preliminary Assessment: Drug Release and Cytotoxicity

In view of the bone tissue engineering applications, the SrRAN release was investigated in phosphate-buffered saline (PBS, pH = 7.2–7.6, Sigma-Aldrich, St. Louis, MO, USA) and in a high-glucose Dulbecco’s modified eagle medium (DMEM, Thermo Fisher Scientific, Waltham, MA, USA). Five cold-sintered pellets (6 mm in diameter) of cold-sintered HAp10ChitSrRAN and three HAp10Chit cold-sintered pellets (as controls) were immersed in 5 mL PBS and DMEM, and incubated at 37 °C in a Grant Instruments™ (Shepreth, Cambridgeshire, UK) Compact Incubator Shaker under continuous shaking (80 rpm) up to 35 days. At each time point, 2 mL of conditioned medium was collected and replaced with 2 mL of fresh medium. The collected medium was then poured into silica cuvettes and analysed at 318 nm wavelength in a Thermo Fisher Scientific (Waltham, MA, USA) Evolution 300 UV–Vis spectrophotometer. A calibration curve was constructed based on five SrRAN standard solutions (80 μg/mL, 40 μg/mL, 9.6 μg/mL, 3.2 μg/mL and 0.8 μg/mL), prepared by dissolving the drug in PBS and/or in DMEM in an ultrasonic bath for 10 min at room temperature and then by magnetic stirring at 600 rpm for 40 min.

The cytotoxicity was also preliminarily assessed by an indirect method according to the ISO 10993-5:2009 standard [83] using an MG63 cell line and the cell counting kit-8 (CCK-8, Sigma Aldrich, St. Louis, MO, USA). After being sterilised in a Tuttnauer (Breda, Noord-Brabant, The Netherlands) Elara 11 autoclave set at 121 °C for 20 min, three replicate samples from each batch were immersed in 2.4 mL cell medium and placed in the incubator at 37 °C under continuous shaking (80 rpm). The cell medium was prepared by mixing DMEM with 10 vol% fetal bovine serum (Gibco, USA) and 1 vol% penicillin/streptomycin (Gibco™, Thermo Fisher Scientific Inc, Waltham, MA, USA). At each time point, 1 mL of conditioned medium was collected and replaced with 1 mL of fresh cell medium. Then, the conditioned medium was sterilised with a 0.22 μm filter and used as a cultured medium in three concentrations (100%, 10% and 1%) for human osteosarcoma cells (MG63). A total of 10,000 MG63 cells were seeded in a 96-well plate and incubated with the conditioned medium (at the three concentrations) for 24 h, 48 h and 72 h in an incubator at 37 °C with 5% CO_2_. The negative and positive control consisted of cells in cultured medium and cells in cultured medium treated with 5% dimethylsulfoxide (DMSO), respectively. The absorbance was then measured with a Tecan (Männedorf, Switzerland) Infinite^®^ 200 PRO microplate reader, set at 450 nm.

The data collected from the preliminary in vitro assessment are reported as mean ± standard deviation. The statistical analysis of the drug release data was carried out by two-way ANOVA test followed by Sídák’s multiple comparison test, and the cytotoxicity test results were analysed by two-way ANOVA and Tukey’s multiple comparison test. 

## 3. Results and Discussion

### 3.1. Dissolution–Precipitation Synthesis of HAp and HAp Composites

**Figure 2:**
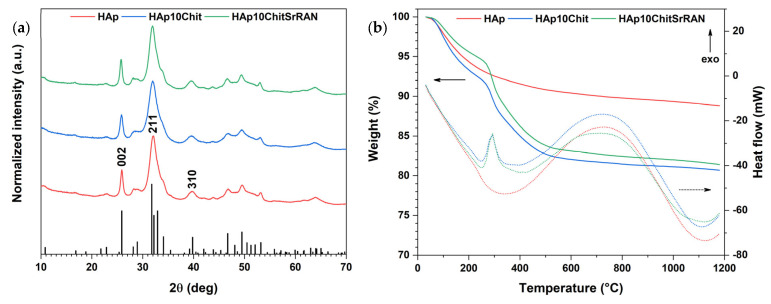
(**a**) XRD patterns of HAp and HAp-based composite powders. The HAp reference pattern corresponds to No. 00-064-0738 in the ICDD database; (**b**) TGA-DSC curves of HAp and HAp-based composite powders. The solid lines represent the weight % of the TGA curves, while the dotted lines represent the heat flow (in mW) of the DSC curves.

The synthesised powders underwent mineralogical investigation by XRD, as shown in Figure 2a. All the patterns matched the reference HAp X-ray diffractogram. Although peak shifts could be expected in the composite materials due to the addition of chitosan and SrRAN, such shifts were comparable with the instrument resolution (0.04°), and thus, were considered as not significant. An evident peak broadening is visible in the XRD patterns. This could result from the crystal disorder [69,84] and the crystallite nanometric size of the produced powders. In any case, the various findings obtained in the present research indicate that the produced powders correspond to nanocrystalline apatite.

Based on the SSA and the measured density reported in Table 1, the powders were found to have an average particle size of ~20 nm, assuming spherical particles. Such a value is actually in good agreement with the crystallite size calculated with Scherrer’s formula, where the 002 and 310 peaks were considered for the crystallite length and width/thickness, respectively, as suggested by Eichert et al. [14]. A similar crystallite size was found for hydroxyapatite powder synthesised by dissolution–precipitation [38] and by wet precipitation [69,84]. 

**Figure 3:**
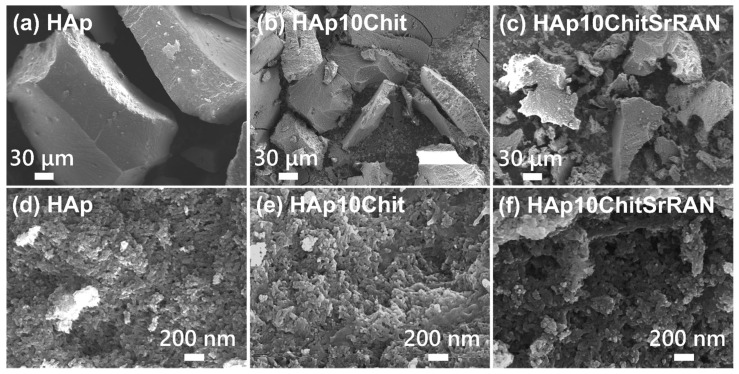
SEM images of HAp (**a**,**d**), HAp10Chit (**b**,**e**) and HAp10ChitSrRAN (**c**,**f**) powder.

The nanometric size of the crystals could hardly be individually observed by scanning electron microscopy. The SEM micrographs in Figure 3a–c, show large particles of tens of micrometres corresponding to highly agglomerated nanocrystals. Agglomerates of nanometric particles with irregular and isometrical shape could be distinguished at higher magnification (Figure 3d–f). 

As for the XRD patterns, the organic materials (i.e., chitosan and strontium ranelate) could not be clearly identified in the SEM images. The presence of organic matter is undoubtedly confirmed by the thermal analyses shown in Figure 2b. In fact, the total weight loss of HAp powder was ~11%, due to the loss of adsorbed and structural water but also of carbonates, as previously reported [55]. Conversely, HAp10Chit and HAp10ChitSrRAN both lost ~19% in total and showed an additional weight loss at ~300 °C in comparison with the HAp thermogravimetric curve, which corresponds to the thermal degradation of the organic matter, as previously reported by Zima [30] and Luo et al. [85]. The mass loss at 300 °C is consistent with an exothermic peak in the DSC curve of composite materials that is associated with the organic matter thermal degradation. An additional exothermic phenomenon occurred between 500 °C and 1100 °C, which could result from the evolution of carbonates, the crystallisation of possible residual amorphous calcium carbonate and apatite in β-tricalcium phosphate [86].

**Figure 4:**
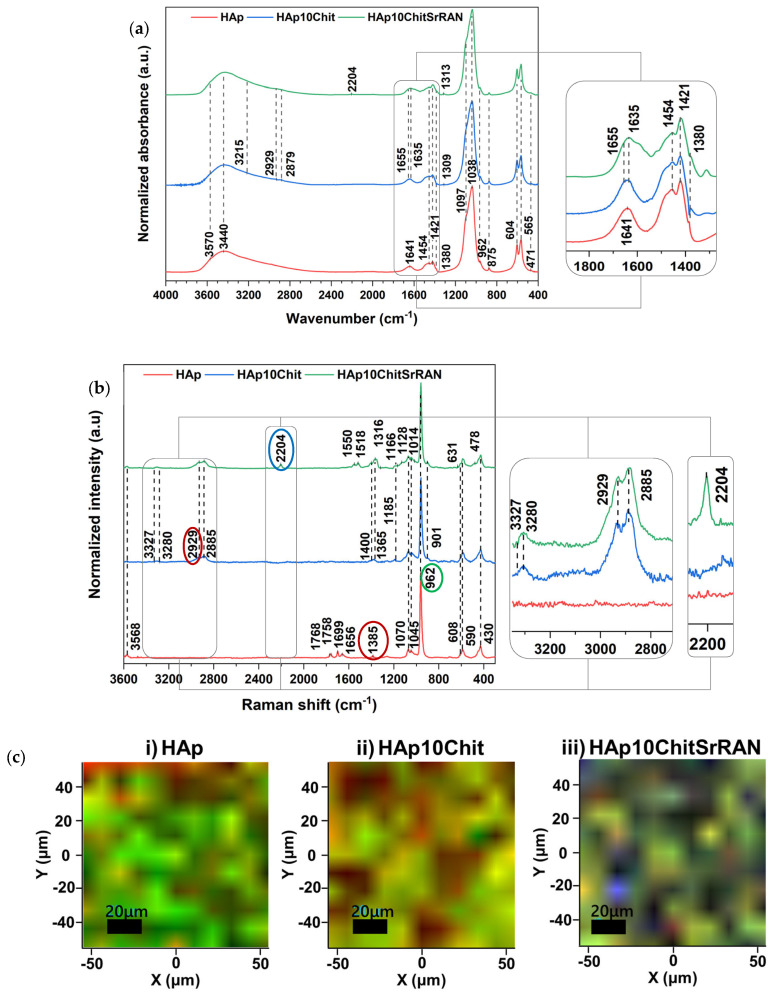
(**a**) FTIR and (**b**) Raman spectra of HAp, HAp10Chit and HAp10ChitSrRAN powders; (**c**) Raman maps indicating the distribution of apatite (green) corresponding to the ν_1_PO_4_ peak at 962 cm^−1^, shell organics (red) corresponding to the N–H peak at 1385 cm^−1^, chitosan (red) corresponding to the C–H peaks at 2929 cm^−1^ and SrRAN (blue) corresponding to the C≡N peak at 2204 cm^−1^.

The presence of organic materials can be further discriminated in the FTIR and Raman spectra in Figure 4a,b, respectively. In particular, chitosan can be identified by its typical infrared signals such as the N–H stretching mode at 3215 cm^−1^, the C–H symmetric and asymmetric stretching mode at 2880–2930 cm^−1^, the amide type I C=O signal at 1654 cm^−1^ and the C–O stretching at 1310 cm^−1^; conversely, SrRAN is identified by the C≡N peak at 2204 cm^−1^. The typical HAp peaks appear in all the infrared spectra, consisting of the phosphate group signals at 1097 cm^−1^ and 1035 cm^−1^ (ν_3_), 962 cm^−1^ (ν_1_), 604 cm^−1^ and 565 cm^−1^ (ν_4_) and at 430 cm^−1^ (ν_2_), and the OH peak at 3570 cm^−1^, as well as bands at 3440 cm^−1^ (ν_1_) and at 1641 cm^−1^ (ν_2_) due to adsorbed and structural water [14]. Moreover, carbonate bands are found at 1454 cm^−1^ and 1421 cm^−1^ (ν_3_), and 875 cm^−1^ (ν_2_), which correspond to a B-type carbonate HAp [15,30,38,87]. Infrared spectroscopy is useful to investigate chemical interactions between hydroxyapatite, chitosan and strontium ranelate. In this scenario, hydrogen bonds, ionic and/or polar interactions were previously reported in the literature [11,88,89,90]. The amide type I C=O peak can be detected at 1655 cm^−1^ in the composite powders and is slightly blue-shifted in comparison with the typical C=O signal in pure chitosan, found at 1660 cm^−1^ [30], possibly due to a hydrogen bond interaction between the OH groups of HAp and the NH_2_ group of chitosan [11,88]. In addition, the bending mode of adsorbed water of HAp at 1641 cm^−1^ is red-shifted to 1635 cm^−1^ in the composite powders, as shown in the zoomed detail of Figure 4a. Raman spectroscopy is more sensitive for identifying chitosan and SrRAN; in fact, chitosan peaks are found at 3330 cm^−1^ due to OH stretching of hydrogen bonds [91], at 3280 cm^−1^ due to N–H stretching, at 2930 cm^−1^ and 2885 cm^−1^ due to C–H stretching of CH_3_ and CH_2_ groups, respectively, and at 1400 cm^−1^ and 1365 cm^−1^ associated with C–H in-plane bending vibrations. Nonetheless, the C–O–C stretching signal appears at 1185 cm^−1^, while the peak at 901 cm^−1^ can be associated with the pyranoid ring stretching of CH_2_ [91,92]. Additional SrRAN signals, other than the C≡N band at 2204 cm^−1^, appear at 1550 cm^−1^ and 1518 cm^−1^ due to amide type II, at 1316 cm^−1^ associated with CH_2_ wagging, at 1166 cm^−1^ and 1128 cm^−1^ due to the C–O asymmetric vibration, at 1014 cm^−1^ due to C–C symmetric stretching and at 631 cm^−1^ associated with C–S signal. In all Raman spectra (Figure 4b), HAp is associated with the OH peak at 3568 cm^−1^ (ν_1_) and 1656 cm^−1^ (ν_2_), and the phosphate bands at 1070 cm^−1^ (ν_3_), 1045 cm^−1^ (ν_3_), 962 cm^−1^ (ν_1_), 608 cm^−1^ and 590 cm^−1^ (ν_4_), and 430 cm^−1^ (ν_2_) [38]. In fact, the ν_3_PO_4_ signal at 1070 cm^−1^ could also be ascribed to a B-type carbonate mode. Peaks appearing at 1770–1700 cm^−1^ and at 1385 cm^−1^ correspond to C=O stretching and N–H vibration, respectively, and they could be due to the presence of residual organic matter from the mussel shells. However, the shell organic matter residuals are not uniformly distributed in the material but mineralised in clusters, as demonstrated by Raman maps in Figure 4(ci). The Raman maps were acquired to investigate the distribution of the organic and inorganic species within the produced powders. In particular, the map of HAp (Figure 4(ci)) was obtained considering the characteristic ν_1_PO_4_ band of apatite at 962 cm^−1^ (green colour in the map) and the band at 1385 cm^−1^ characteristic of the organic matter of the shell (red colour in the map). The maps of the composite powders (Figure 4(cii,ciii)) were instead acquired considering the C≡N of SrRAN at 2204 cm^−1^ (blue colour), the νCH of chitosan at 2929 cm^−1^ (red colour) and the ν_1_PO_4_ band at 962 cm^−1^ for apatite (green colour), as indicated by the colourful circles in the Raman spectra in Figure 4b. Chitosan and SrRAN are found evenly spread within the apatite powder, resulting in purple spots due to the superimposition of the blue spots of SrRAN and the red spots of chitosan and shell organics. The good distribution of polymer and the drug further demonstrates the advantage of the dissolution–precipitation method in producing homogeneous systems in comparison with simply dry-pressing [93]. 

The synthesised powders were further subjected to elemental analyses, and the results are reported in Table 2. The carbonate content in the produced powders is around 3 wt% in the typical range for bone apatite (2–8 wt% [15,87]), thus validating the previous hypothesis concerning the production of carbonated hydroxyapatite. Traces of Na, Mg and Sr are also identified in all samples due to the use of mussel shells as biogenic raw material. The larger amount of Sr in the HAp10ChitSrRAN is a further confirmation of the presence of SrRAN in the material. Ion substitution is a typical feature of bone apatite, which represents a reservoir of alkaline and alkaline-earth metal ions playing fundamental roles in several bone biological functions, as investigated in-depth in the literature [10,11,94,95]. For example, Na ions substitution in HAp (NaHAp) affects cell adhesion, bone metabolism and regeneration. In particular, Sang Cho et al. [96] reported improved osteoconductivity of NaHAp with respect to pure HAp considering a calvarial defect model in New Zealand white rats. Magnesium is another essential substitutional ion in natural apatite and is actively involved in bone mineralisation and remodelling by stimulating osteoblast proliferation [97], whereas a deficiency of Mg facilitates osteoporosis and osteopenia due to the enhanced proliferation of osteoclasts [9,97]. Landi et al. [98] observed a superior adhesion and proliferation both of mesenchymal stem cells and of osteoblastic-like cells (MG-63) in comparison with a synthetic non-substituted hydroxyapatite. In addition, strontium is a well-known substitutional element able to sustain mineralisation and bone remodelling by impairing osteoclast/osteoblast activities; particularly, Sr promotes bone formation by osteoblasts and hinders bone resorption by osteoclasts. Stipniece et al. [99] produced a Sr-substituted hydroxyapatite (SrHAp) with strontium content between 1 wt% and 8 wt% and observed that Sr directly triggered and accelerated the maturation of primary human osteoblast cells into osteocytes in comparison with pure HAp. An enhanced proliferation of osteosarcoma cells (SAOS-2) on SrHAp was observed by Frasnelli et al. [100] considering a non-substituted HAp as a control, while the cell morphology was practically unchanged by the different concentration of Sr. 

Interestingly, the amount of HPO_4_ ions is higher in the composites (0.26 wt%) than in HAp (0.15 wt%). Such a finding could suggest that the interactions between HAp and chitosan involve the surface hydrated layer. As reported in Table 2, the Ca/P ratio of HAp is 1.38 well below the stoichiometric Ca/P of 1.67, indicating a calcium-deficient apatite and supporting the hypothesis of a non-stoichiometric, multi-ion-substituted apatite with limited crystallinity. In some previous studies, Ortali et al. [101] reported a Ca/P ratio of ~1.35 for a calcium-deficient HAp and of ~1.33 for a biomimetic nanocrystalline apatite, whereas Drouet et al. [20] observed a gradual increase in the Ca/P ratio of nanocrystalline apatite from 1.30 to 1.48, depending on the maturation time, which was up to 20 days. The Ca/P ratio in the composites further decreases to 1.26 for HAp10Chit and to 1.27 for HAp10ChitSrRAN. These obtained values are not statistically different but they suggest that chitosan could interact with apatite and prevent or limit its crystallisation. 

**Figure 5:**
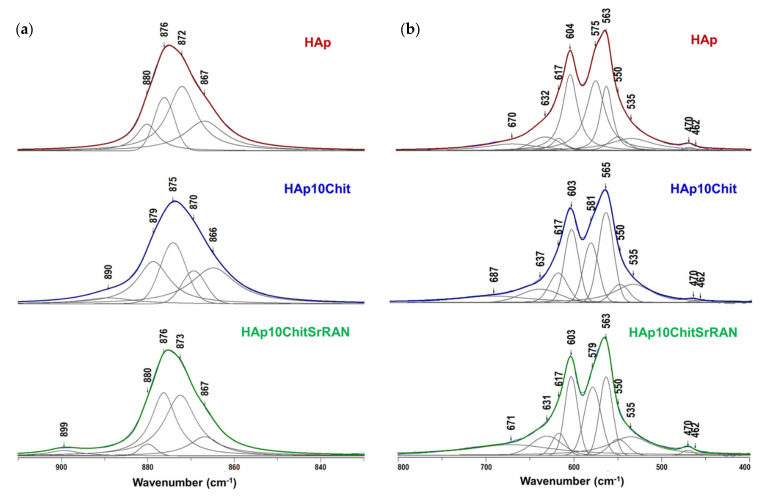
Decomposition of carbonates (**a**) and phosphates (**b**) band domains of the FTIR spectra of HAp, HAp10Chit and HAp10ChitSrRAN synthesised powders.

The non-stoichiometry of the produced apatite powder was further investigated by peak decomposition of the ν_4_PO_4_ (800–400 cm^−1^) and ν_2_CO_3_ (900–800 cm^−1^) infrared spectral domains. Indeed, in the case of biomimetic apatite, these spectral domains are characterised by the presence of apatitic and non-apatitic phosphate and carbonate environments [21,74,84,101]: the apatitic ones include ions in the apatite normal crystallographic sites, while the non-apatitic ions are not located within the apatite lattice [20]. As a matter of fact, the non-apatitic (or labile) environments in bone-like apatite are associated with a thin hydrated layer surrounding the nanocrystals, where exchangeable ions (mostly divalent Ca^2+^, HPO_4_^2−^ and CO_3_^2−^) are present [21,74,84,101]. Due to the metastability of bone-like apatite, upon maturation and/or heat treatment, the hydrated layer could gradually disappear and the labile ions could be incorporated into the bulk apatite domains, thus resulting in a more stable apatite with a higher crystallinity and stoichiometry closer to 1.67 [13]. Indeed, fully crystalline stoichiometric HAp does not have non-apatitic environments. In the present study, the ν_2_ and ν_4_ phosphate domain decomposition in Figure 5b was carried out by imposing several constraints for the fitting, as previously reported in other investigations on biomimetic apatites [84,102]: fixed peak positions at 617 cm^−1^, 550 cm^−1^ and 535 cm^−1^; a fixed width of 25 cm^−1^ for the apatitic HPO_4_^2−^ peak at 550 cm^−1^ and of 15 cm^−1^ for the ν_2_PO_4_ peak at 470 cm^−1^; and a Lorentzian curve shape for all peaks except for the Gaussian peak at ~630 cm^−1^. The non-apatitic PO_4_ and HPO_4_ bands can be observed in all the produced powders at 617 cm^−1^ and 535 cm^−1^, respectively. The ν_4_ mode of apatitic PO_4_ and HPO_4_ groups is instead associated with the bands at ~603 cm^−1^, 575 cm^−1^, ~563 cm^−1^ and 550 cm^−1^, while the ν_2_ mode is found at 470 cm^−1^ and 462 cm^−1^. 

**Figure 6:**
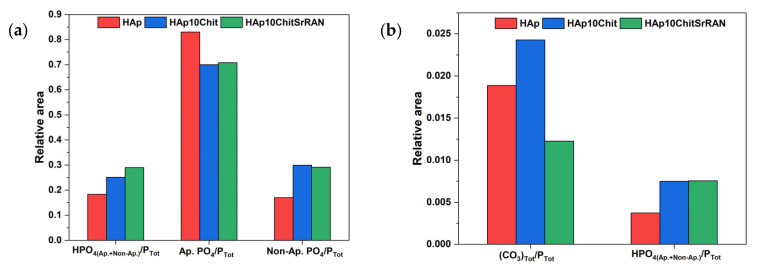
Relative area of peaks resulting from the decomposition of ν_4_PO_4_ (**a**) and ν_2_CO_3_ (**b**) domains of HAp (red), HAp10Chit (blue) and HAp10ChitSrRAN (green) powder. (Ap. = apatitic; Non-Ap. = non apatitic).

In addition, the OH libration band at ~632 cm^−1^ and the H_2_O band at ~670 cm^−1^ are observed. The decomposition of the ν_2_CO_3_ domain (Figure 5a) was carried out by imposing the A-site CO_3_ band at ~878 cm^−1^, the B-site CO_3_ band at ~872 cm^−1^ and the non-apatitic CO_3_ band at 866 cm^−1^, based on the previous study by Ortali et al. [74]. The carbonate decomposition of all the powders is characterised by an additional band at ~876 cm^−1^ due HPO_4_, apatitic and non-apatitic. The Hap10Chit and Hap10ChitSrRAN spectral domains show additional bands at 890 cm^−1^ and 899 cm^−1^, respectively, due to the C–O–C signal of chitosan [30]. These results confirm the production of a carbonated apatite even in the presence of chitosan and SrRAN. According to the spectral decomposition of the PO_4_ and CO_3_ domains, the contribution of the apatitic and non-apatitic environments can be evaluated by considering the relative area of the peaks. As reported in Figure 6, the amount of HPO_4_ ions is larger in the composites than in the HAp, in agreement with the elemental analysis in Table 2. Also, the contribution of non-apatitic peaks increases in the composites in comparison with HAp, which suggests that the interaction between HAp and chitosan involves the hydrated layer (Figure 6a) and it might justify the lower Ca/P ratio of the HAp/chitosan composites (Table 2). The analysis of the CO_3_ domain is more complex due to the overlapping of several bands and some bias cannot be excluded since the domain contains many peaks in a narrow area. Indeed, HAp10ChitSrRAN shows a lower content of carbonates in comparison with HAp, in line with the elemental analysis, but the relative area of CO_3_ in HAp10Chit appears overestimated in Figure 6b in comparison with the elemental analysis results, where the amount of carbonate in HAp10Chit is slightly lower than in HAp (Table 2). Finally, the relative area of HPO_4_ calculated on the ν_4_PO_4_ domain data and on the HPO_4_ band within the ν_2_CO_3_ domain (at 876 cm^−1^) are slightly different, but the trends between the samples are consistent.

On the basis of the results reported so far, the synthesised HAp- and HApChit-based composites are examples of biomimetic apatites due to their nanocrystallinity, content in non-apatitic phosphate and carbonate groups, ion substitution and non-stoichiometry.

### 3.2. Cold Sintering of HAp and HAp-Based Composites

**Figure 7:**
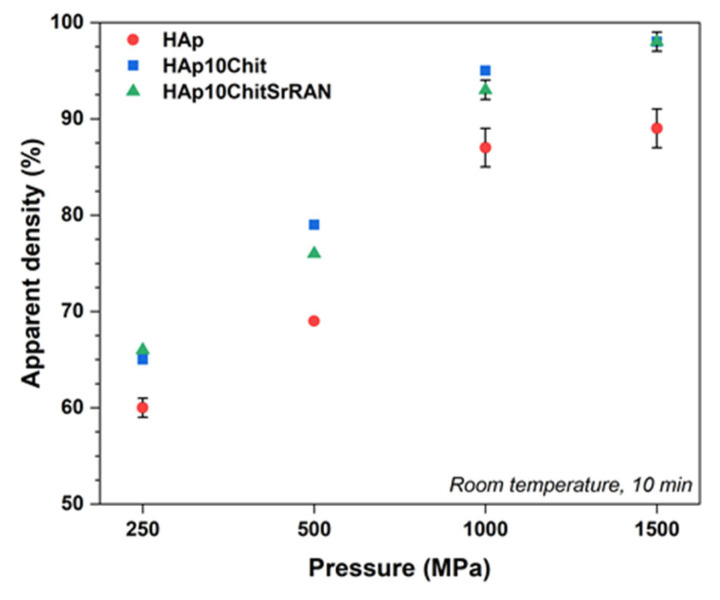
Relative apparent density of cold-sintered HAp, HAp10Chit and HAp10ChitSrRAN. The cold-sintered pellets were produced at room temperature by holding the applied pressure for 10 min.

The produced powders were transformed into bulk monoliths by cold sintering at room temperature and without any transient liquid phase. As shown in Figure 7, by increasing the applied pressure from 250 MPa to 1500 MPa, the relative density of the obtained pellets consequently increased. The relative density, which was calculated considering the density of the HAp, HAp10Chit and HAp10ChitSrRAN powders measured by He pycnometry, was equal to 2.68 ± 0.02 g/cm^3^, 2.43 ± 0.01 g/cm^3^ and 2.45 ± 0.01 g/cm^3^, respectively (see Table 1). It is interesting to highlight that the relative density of HAp10Chit and HAp10ChitSrRAN samples was higher than that of the HAp pellets. This finding could suggest that the hydrated layer plays an effective role in the consolidation of the material, causing the composites to have a larger amount of non-apatitic environments (Figure 6), as previously hypothesised by Ortali et al. [74,101].

**Figure 8:**
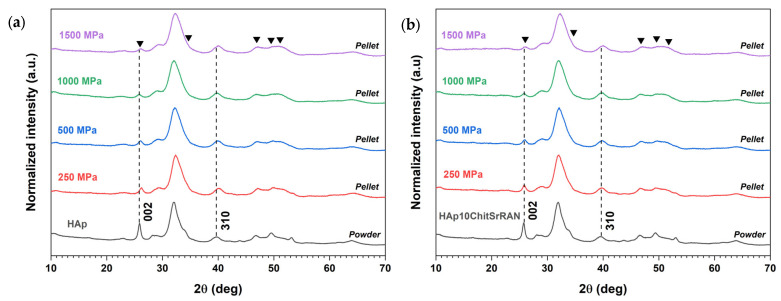
XRD patterns of HAp (**a**) and HAp10ChitSrRAN (**b**) pellets cold-sintered at room temperature under pressure from 250 MPa to 1500 MPa and 10 min holding time. The black triangles indicate the main peaks undergoing modifications after cold sintering.

The apatite nanocrystalline phase was retained in all sintered samples independently of the applied pressure, as demonstrated by the XRD patterns of the HAp and HAp10ChitSrRAN samples in Figure 8 and Figure S1.

Although no apatite phase transformation can be detected after cold sintering, one can point out a change in the relative intensity of the 002 peak and a broadening of all the peaks, which appear less sharp and defined for the pellets compared with the corresponding initial powders as the applied pressure increases. On the other hand, relative intensity and shape modification of the XRD peaks are less pronounced in the diffraction patterns for the crushed pellets (Figure S1). Therefore, a texture effect can be excluded as a consequence of the intense applied external pressure. Instead, the different relative intensities and shapes of the diffraction peaks after cold sintering can be ascribed to the deformation and orientation of the crystals in the very first layer of the sample surface due to the applied pressure, whereas the crystals within the pellet are less affected by the mechanical stress. This hypothesis agrees with the well-known pressure gradient/distribution typical of pressure-based forming and sintering processes due to the uneven transmission of the applied load (such as dry or wet pressing, hot pressing, etc.) [66,103]. In most previous works on cold sintering, the material retains its crystallographic structure with bare modification of peak intensity and/or shape after consolidation, although a few exceptions are also reported; for example, Grossin et al. [21] showed a gradual peak broadening by increasing the applied pressure from 5 MPa to 50 MPa to 100 MPa during spark plasma sintering of biomimetic apatite. Also, Nayir et al. [104] observed a preferred orientation along the (002) plane in MoS_2_ pellets in comparison with MoS_2_ raw powder and ascribed this phenomenon to the mechanical stress applied during the sintering process.

**Figure 9:**
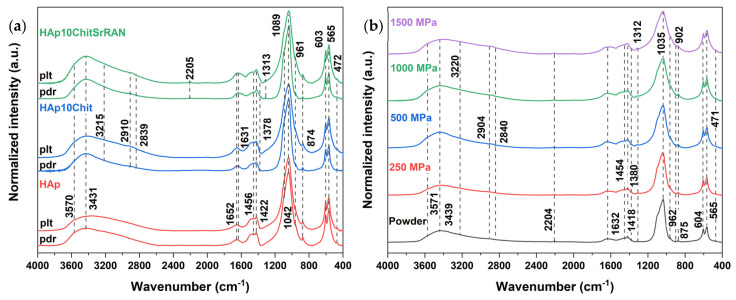
(**a**) FTIR spectra of cold-sintered pellets (plt) at 1500 MPa in comparison with raw powders (pdr); (**b**) FTIR spectra of cold-sintered HAp10ChitSrRAN pellets pressed at 250 MPa, 500 MPa, 1000 MPa and 1500 MPa.

The effect of pressure was further evaluated by FTIR and Raman spectroscopy, as shown in Figure 9 and Figure S2a,b. Interestingly, none of the three materials under investigation showed appreciable shifts in infrared or Raman signals after being cold-sintered, independently of the applied pressure, highlighting the great potential of cold sintering for the consolidation of metastable materials. According to Rey et al. [105], pressure effects on crystals can be observed in vibrational spectroscopy (i.e., Raman and FTIR) and involve signal shifts depending on the vibrational mode, but only when extreme pressure levels are applied. In the case of fluorapatite, for example, the ν_4_, ν_1_ and ν_3_ phosphate bands shift from 550/600 cm^−1^ to 600/650 cm^−1^, from 960 cm^−1^ to 1050 cm^−1^ and from 1000/1100 cm^−1^ to 1100/1200 cm^−1^, respectively, when the applied pressure gradually increased from 1.5 GPa to 24.9 GPa [106]. In the current work, vibrational signal shifts under pressure cannot be excluded but they are most likely reversible phenomena, disappearing once the mechanical load is released. In addition to band shifting, peak broadening can also result from pressure increase [105], as recently reported by Le Grill et al. [107] for the consolidation of amorphous calcium phosphate under ultrafast compression. Indeed, the ν_1_ and ν_3_ phosphate vibrational bands in Figure 9b (clearer in Figure S2c) undergo broadening for pressure increasing from 250 MPa to 1500 MPa, while carbonate bands are less affected by pressure because of the molecular trigonal planar geometry of carbonate groups in comparison with the tetrahedral geometry of phosphate groups, which is in agreement with the pressure effect studies on carbonated apatites by de Carmejane et al. [108].

Nonetheless, the decomposition of the FTIR bands in the ν_2_CO_3_ (910–830 cm^−1^) domain, also including the HPO_4_ band and ν_2_ν_4_PO_4_ (800–400 cm^−1^) domains of the cold-sintered pellets pressed at 1500 MPa shown in Figure S3, is quite similar to the FTIR band decomposition of the produced powders. In addition, the relative area of the HPO_4_ band, the apatitic and non-apatitic environments in the PO_4_ domain in Figure S4b have the same trend as observed in the FTIR band decomposition of the powder spectra. Conversely, the relative area of HPO_4_ at ~876 cm^−1^ is larger for HAp than for the composites’ spectra but as previously mentioned, the narrow area of analysis could be a source of bias.

**Figure 10:**
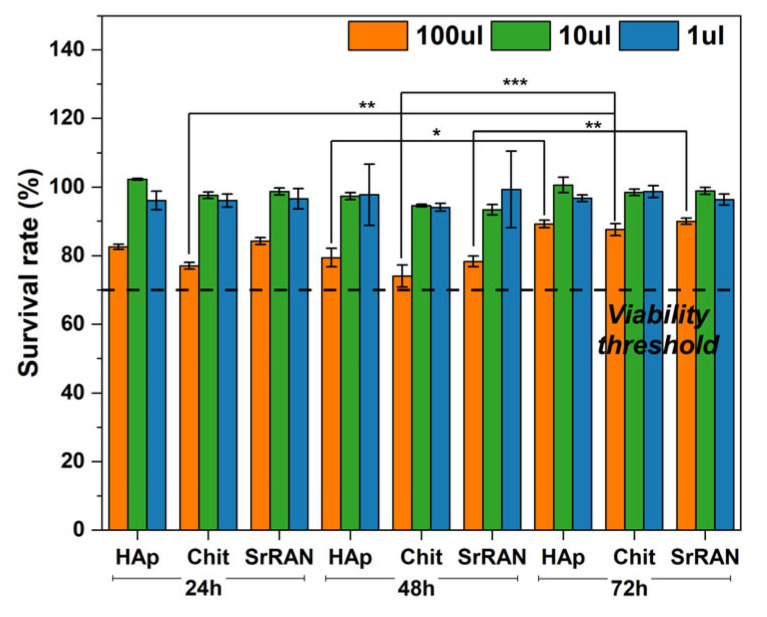
Cytotoxicity assessment of HAp, HAp10Chit and HAp10ChitSrRAN cold-sintered pellets by indirect test according to the ISO 10993-5:2009 standard: survival rate at 24 h, 48 h and 72 h of MG63 cells in contact with various volume of extract (1, 10 or 100 μL). The black dotted line represents the viability threshold. (*: *p*-value < 0.05, **: *p*-value < 0.005, ***: *p*-value < 0.001).

As shown in Figure 10, the release of SrRAN was tested in PBS in physiological conditions (pH~7.4 at 37 °C) and DMEM to better mimic the complexity of body fluids, being used as a basal medium for cell culture. The drug release was gradually sustained for about 19 days in DMEM and for more than 35 days in PBS, without any burst effect in either medium. The release rate can be evaluated by considering the time required to release 50% of SrRAN; accordingly, 4 days were required to release ~50% of the cargo in DMEM, in contrast to the 7 days necessary in PBS. The difference in release rates is confirmed by the performed statistical analysis and can be associated to the different medium composition, material solubility and ion diffusion [109,110]. In fact, both media contain phosphate ions, which can interact with the HAp/chitosan sample, but DMEM is also enriched with amino acids and vitamins which might play a role in the release kinetics, as previously observed by Ramana et al. [111]. Moreover, swelling and open porosity could also influence the diffusion mechanisms governing SrRAN release. Indeed, the HAp10ChitSrRAN cold-sintered pellets have a ~90% relative density, as shown in Figure 7, and HAp10Chit pellets have ~2–5% open pores and show ~10% swelling once immersed in simulated body fluid (SBF), according to our previous investigation on HAp/chitosan composites [55]. According to the literature, the SrRAN concentration used for antiosteoporotic therapy ranges between 0.05 mM and 4 mM, considering both the systemic and local administration [61,112]. Guo et al. [112] suggested that the 0.25 mM–0.5 mM dose range is the most effective for local delivery systems to promote osteogenesis and angiogenesis on ovariectomy rat bone marrow mesenchymal stem cells and human umbilical vein endothelial cells, respectively. However, these authors also pointed out that the effective dose could be affected strongly by the cell line and culture medium used for the investigation. In the present study, the released cargo concentration was ~20 mg/mL (~0.04 mM). Moreover, it is desirable to design the SrRAN-based therapy as a long-term strategy to guarantee high efficacy [60,113]. The current study is one of the first attempts to exploit cold-sintered calcium phosphates for drug delivery applications, and it was observed that cold-sintered HAp/chitosan composite loaded with SrRAN had a burst-free, sustained, gradual and continuous release for 19 days in DMEM and more than 35 days in PBS. Future studies should improve the drug loading concentration and efficiency, and the release rate. In particular, it could be of interest to modulate the porosity/density of the sintered body depending on the drug and on the desirable therapeutic release and concentration. For example, Hu et al. [31] recently investigated the release of doxorubicin (DOX) by immersing DOX-loaded HAp/polylactic acid (HAp/PLA) cold-sintered composites in PBS and showed a faster drug release by increasing the PLA content in comparison with pure HAp. Luginina et al. [114] also considered the possibility of drug delivery applications for spark plasma sintered ACP in light of the high reactivity and resorbability of nanometric ACP and of the residual open porosity (~40–55%) after sintering.

**Figure 11:**
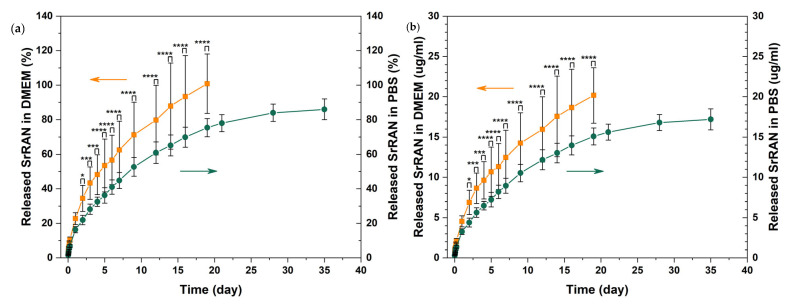
SrRAN release from cold-sintered HAp10ChitSrRAN pellets in DMEM and PBS expressed in % (**a**) and g/mL (**b**). The orange curve represents the release in DMEM, and the green line represents the release in PBS. (*: *p*-value < 0.05, ***: *p*-value < 0.005, ****: *p*-value < 0.0001).

Despite the beneficial effects of SrRAN as an antiosteoporotic agent in promoting osteoblast over osteoclast activity [59,113], controversy in the use of SrRAN has arisen in light of reported cases of heart and cardiovascular diseases as side effects, especially for high drug dosage combined with long term therapy and systemic release [62,115,116]. Therefore, in addition to the drug release test, a preliminary cell viability assessment was carried out here. After sterilization, the samples did not show significant change in comparison with non-sterile pellets, except for a slight increase in the crystallinity, as reported in Figure S5, and a change in the sample colour (from pale yellow to yellow). This phenomenon could be very likely ascribed to the Maillard reaction, as also reported by Vaz et al. [117]. The limited changes the materials undergo after the sterilisation could be because chitosan is associated with HAp and processed under pressure to form a pellet. This could stabilise chitosan in light of the intimate contact with HAp during cold sintering and the limited (chitosan) surface exposed to steam autoclaving. The cytotoxicity evaluation shows a survival rate above 70% for MG63 cells after 24 h, 48 h and 72 h, as reported in Figure 11. Statistically significant differences were found between the cytotoxicity evaluation after 24 h and 48 h with respect to cytotoxicity results after 72 h of incubation, with cells showing a greater survival rate after 72 h. Therefore, none of the extracts collected from cold-sintered samples immersed in culture medium showed cytotoxic effects, according to the ISO 10993-5:2009 standard, regardless of the extract volume concentration (1, 10 or 100 μL) in contact with MG63 cells. Interestingly, the SrRAN-loaded samples showed levels of cell viability similar to those of the drug-free samples, thus indicating that the amount of SrRAN was not detrimental to cells. Similar findings were reported by Loca et al. [60], who tested the cytotoxicity effect on MG63 cells of SrRAN-loaded microcapsules as long-term local delivery systems. Future in vitro studies such as osteogenic differentiation by alkaline phosphatase activity and matrix mineralisation by Alizarin Red S assay should be performed to verify the effectiveness of the released SrRAN in impairing bone turnover.

## 4. Conclusions

Mussel shell-derived biomimetic apatite/chitosan composite powders loaded with antiosteoporotic drug strontium ranelate (SrRAN) were successfully produced by dissolution–precipitation synthesis. The physicochemical characterisation of the produced materials revealed that the synthesised apatite was nanocrystalline, ion-substituted and had limited crystallinity, thus resembling bone apatite, while both chitosan and SrRAN were proven to be homogeneously distributed within the composite. The composite powders were consolidated above 90% relative density by a quite fast (10 min) cold sintering process at room temperature. The consolidation of the composites could have been promoted by the hydrated layer surrounding the apatite nanoparticles and in particular by the larger amount of non-apatitic environments resulting from the addition of chitosan and SrRAN.

Despite the intense uniaxial pressure (up to 1.5 GPa) applied during cold sintering, the composite did not undergo any modification after consolidation, which is further evidence of the great potential and relevance cold sintering has in the consolidation of metastable and temperature-sensitive materials. Nonetheless, it was shown that cold-sintered apatite/chitosan was able to sustain the SrRAN release for more than 15 days depending on the medium, and the released drug was found to have no cytotoxic effects on MG63 cells, according to the preliminary cell viability test. Although further cellular in vitro tests should be carried out to prove the efficacy of released SrRAN in promoting bone formation, the current study represents a first promising step in the development of functionalised cold-sintered composites for sustained local drug release combined with bone tissue engineering applications.

## Figures and Tables

**Table 1:** SSA measurements by BET, density measured by He pycnometry and average particle size of HAp and HAp-based composites powders produced by dissolution–precipitation synthesis. * The average particle size was estimated, assuming spherical particles. ** The average crystallite length and width/thickness was calculated by the Scherrer formula.

Powders	SSA (m^2^/g)	Measured Density (g/cm^3^)	Average Particle Size (nm) *	Average Crystallite Length (nm) **	Average Crystallite Width/Thickness (nm) **
HAp	138 ± 2	2.68 ± 0.02	16 ± 0	17	6
HAp10Chit	143 ± 4	2.43 ± 0.01	17 ± 1	14	5
HAp10ChitSrRAN	142 ± 2	2.45 ± 0.01	17 ± 0	18	6

**Table 2:** Elemental composition of HAp, HAp10Chit and HAp10ChitSrRAN synthesised powders: wt% of cations obtained by AAS, carbonate by coulometry and phosphate by visible colorimetry.

	HAp	HAp10Chit	HAp10ChitSrRAN
Ca^2+^ (wt%)	32.45 ± 0.02	29.65 ± 0.02	29.04 ± 0.02
P^−^ (wt%)	18.13 ± 0.02	18.25 ± 0.02	17.70 ± 0.02
HPO_4_^2−^ (wt%)	0.15 ± 0.02	0.26 ± 0.02	0.26 ± 0.02
Na^+^ (wt%)	0.34 ± 0.02	0.32 ± 0.02	0.29 ± 0.02
Mg^2+^ (wt%)	0.05 ± 0.02	0.04 ± 0.02	0.05 ± 0.02
Sr^2+^ (wt%)	0.09 ± 0.02	0.08 ± 0.02	1.36 ± 0.02
CO_3_^2−^ (wt%)	3.3 ± 0.2	3.1 ± 0.2	2.6 ± 0.2
Ca/P	1.38	1.26	1.27

## Data Availability

The data presented in this study are available on request from the corresponding author.

## Supplementary Materials

The following supporting information can be downloaded at: https://www.mdpi.com/article/10.3390/nano14050441/s1. Figure S1. XRD of cold sintered pellets: entire (figures on the left) and crushed (figures on the right) of HAp, HAp10Chit and HAp10ChitSrRAN. Figure S2. (a) Raman spectra of cold sintered pellets (plt) at 1500 MPa in comparison to initial raw powders (pdr) of HAp, HAp10Chit and HAp10ChitSrRAN; (b) Raman spectra of cold sintered HAp10ChitSrRAN pellets pressed at 250 MPa, 500 MPa, 1000 MPa and 1500 MPa. (c) FTIR spectra of cold sintered HAp10ChitSrRAN pellets pressed at 250 MPa, 500 MPa, 1000 MPa and 1500 MPa. Broadening of the ν_1_ν_3_ phosphate band is highlighted by the dotted rectangle. Figure S3. Decomposition of CO_3_ (on the left) and PO_4_ (on the right) domains of the FITR spectra of HAp, HAp10Chit and HAp10ChitSrRAN cold sintered pellets pressed at 1500 MPa for 10 min at room temperature. Figure S4. Relative area of peaks resulted from the decomposition of PO_4_ (a) and CO_3_ (b) domains of HAp (red), HAp10Chit (blue) and HAp10ChitSrRAN (green) cold sintered pellets pressed at 1500 MPa for 10 min at room temperature. Figure S5. (a) XRD of HAp10Chit pellet before and after autoclave sterilisation; (b) FTIR of HAp and HAp10Chit pellets before and after autoclave sterilisation.
