# Supplementary material for: Apatite/Chitosan Composites Formed by Cold Sintering for Drug Delivery and Bone Tissue Engineering Applications
Source: Nanomaterials (Basel). 2024 Feb 28;14(5):441. doi: 10.3390/nano14050441 (PMC10934113; doi:10.3390/nano14050441)
Supplement: Supplementary file 1 [file nanomaterials-14-00441-s001.zip › nanomaterials-2867794-supplementary.pdf]

Supplementary material

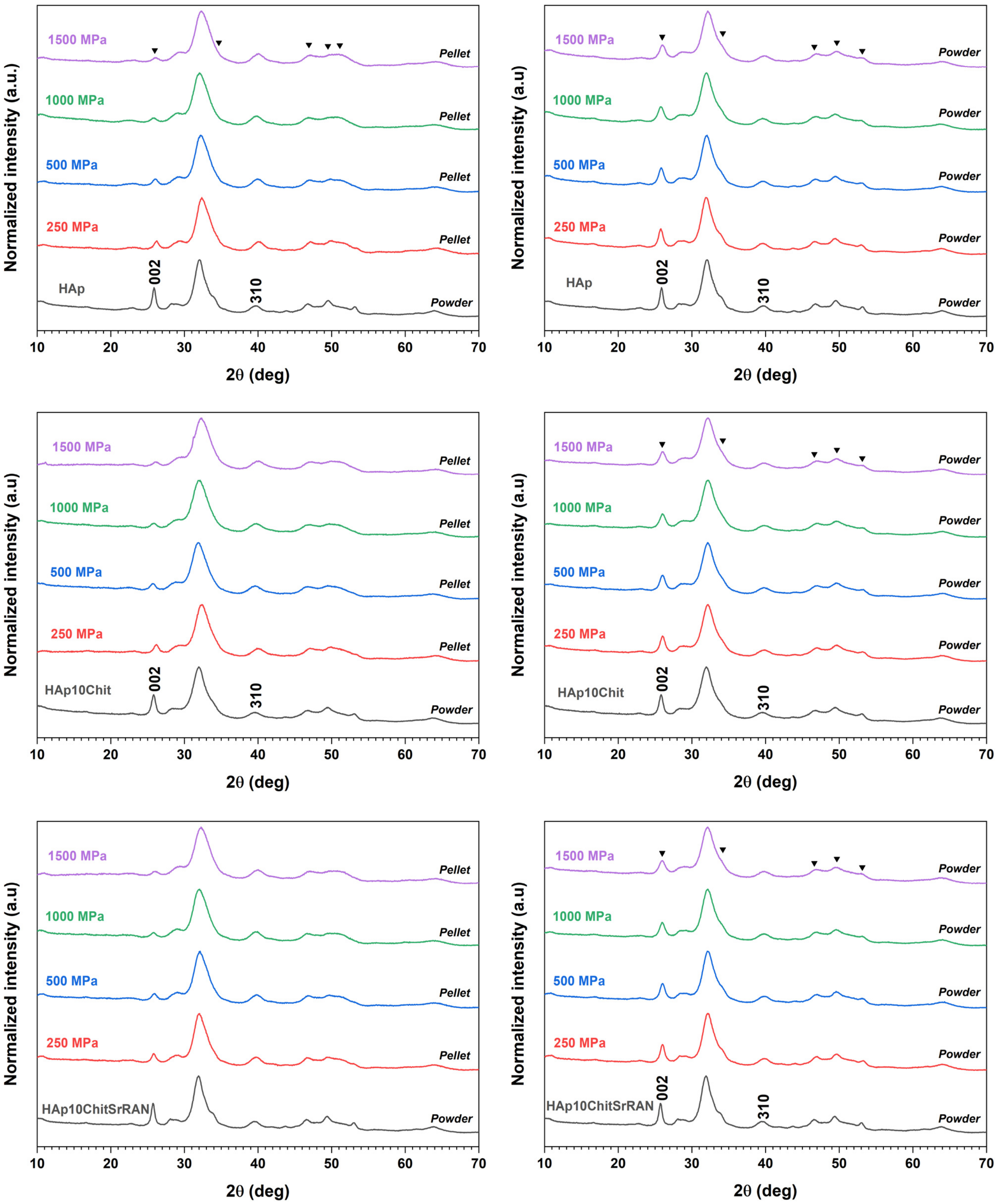

Figure S1 XRD of cold sintered pellets: entire (figures on the left) and crushed (figures on the right) of HAp, HAp10Chit and HAp10ChitSrRAN.

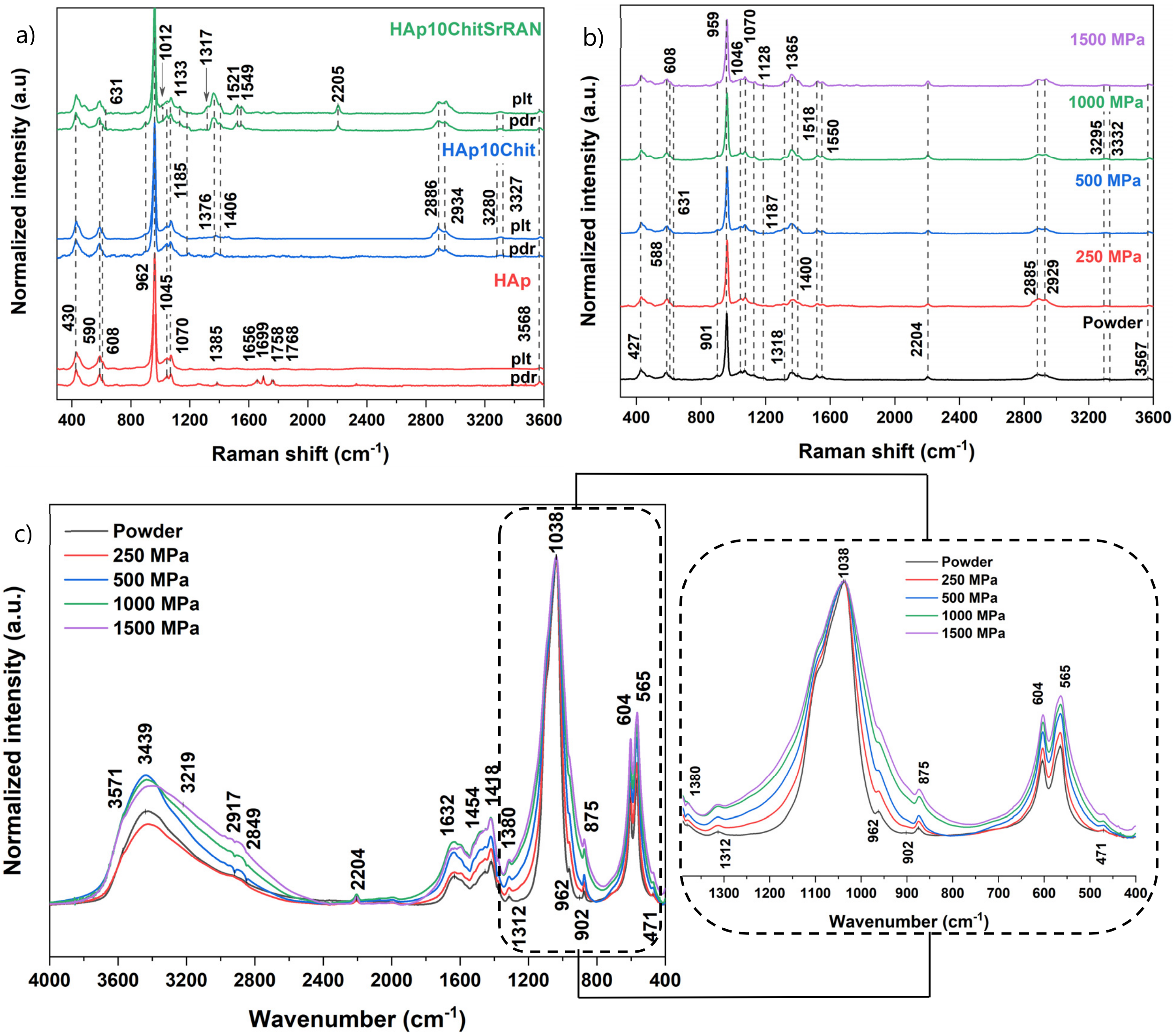

Figure S2 a) Raman spectra of cold sintered pellets (plt) at 1500 MPa in comparison to initial raw powders (pdr) of HAp, HAp10Chit and HAp10ChitSrRAN; b) Raman spectra of cold sintered HAp10ChitSrRAN pellets pressed at 250 MPa, 500 MPa, 1000 MPa and 1500 MPa. c) FTIR spectra of cold sintered HAp10ChitSrRAN pellets pressed at 250 MPa, 500 MPa, 1000 MPa and 1500 MPa. Broadening of the $\nu_1\nu_3$ phosphate band is highlighted by the dotted rectangle.

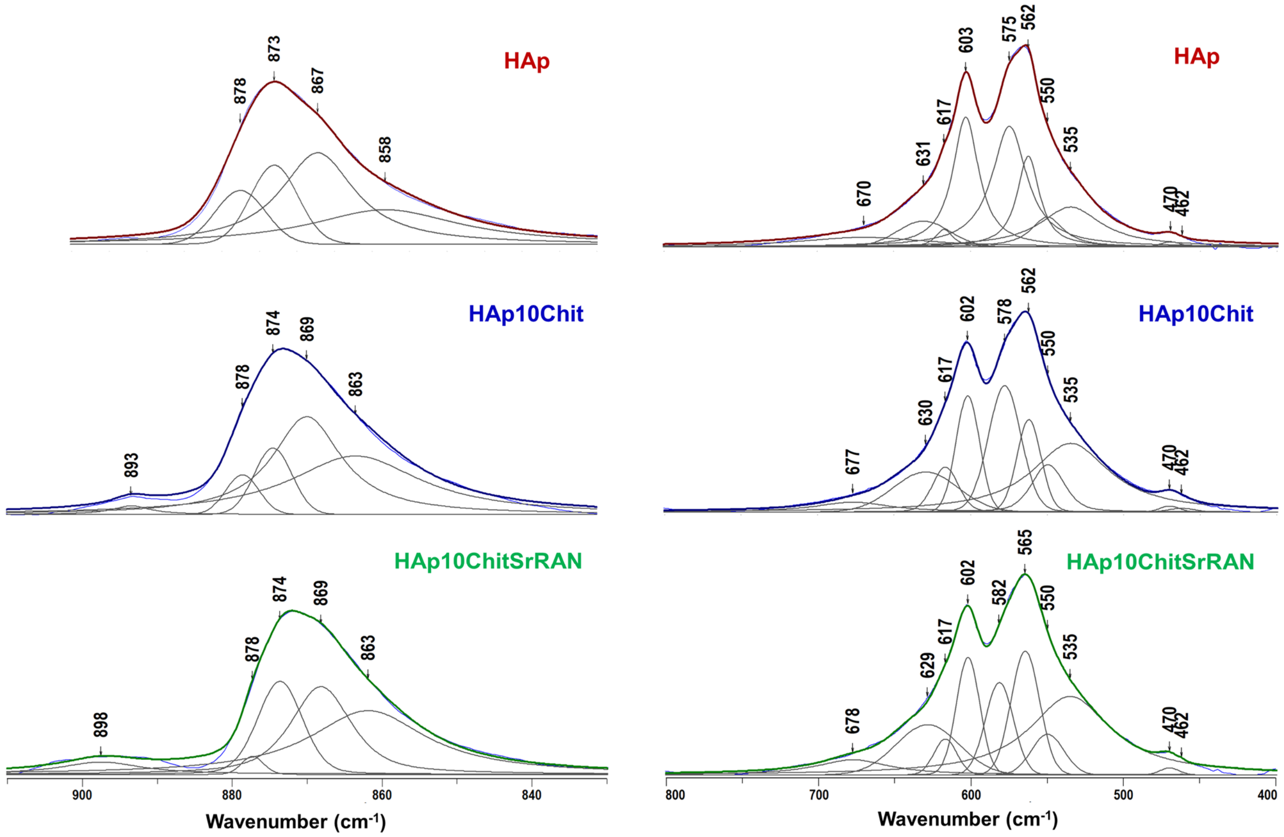

Figure S3 Decomposition of CO_3 (on the left) and PO_4 (on the right) domains of the FITR spectra of HAp, HAp10Chit and HAp10ChitSrRAN cold sintered pellets pressed at 1500 MPa for 10 min at room temperature.

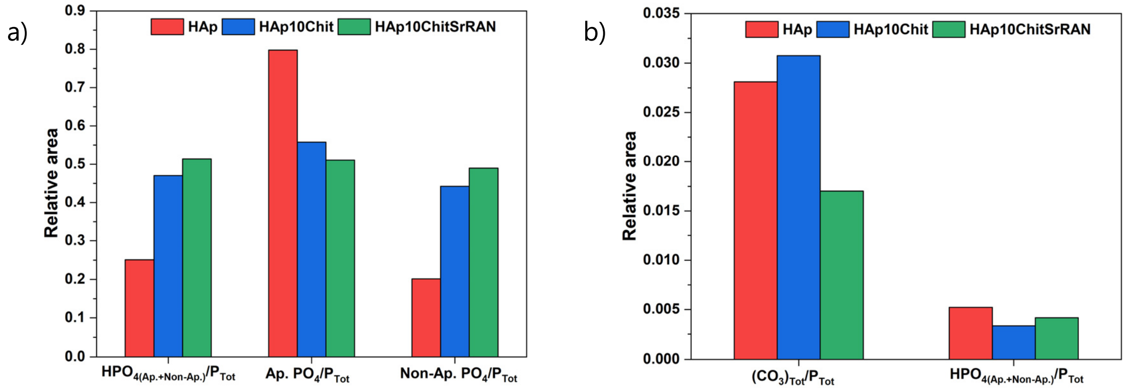

Figure S4 Relative area of peaks resulted from the decomposition of PO_4 (a) and CO_3 (b) domains of HAp (red), HAp10Chit (blue) and HAp10ChitSrRAN (green) cold sintered pellets pressed at 1500 MPa for 10 min at room temperature.

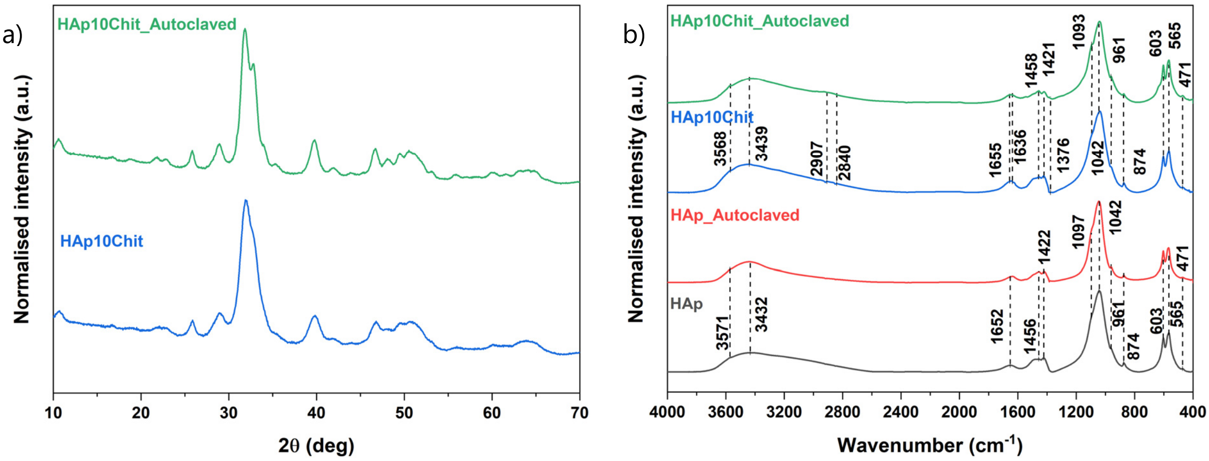

Figure S5 a) XRD of HAp10Chit pellet before and after autoclave sterilisation; b) FTIR of HAp and HAp10Chit pellets before and after autoclave sterilisation.
